# Collaboration between tumor-specific CD4^+^ T cells and B cells in anti-cancer immunity

**DOI:** 10.18632/oncotarget.8797

**Published:** 2016-04-18

**Authors:** Thomas V. Guy, Alexandra M. Terry, Holly A. Bolton, David G. Hancock, Erhua Zhu, Robert Brink, Helen M. McGuire, Elena Shklovskaya, Barbara Fazekas de St Groth

**Affiliations:** ^1^ T Cell Biology Laboratory, Centenary Institute of Cancer Medicine and Cell Biology, The University of Sydney, Sydney, NSW, Australia; ^2^ Discipline of Dermatology, Sydney Medical School, The University of Sydney, Sydney, NSW, Australia; ^3^ B Cell Laboratory, Garvan Institute of Medical Research, Darlinghurst, NSW, Australia

**Keywords:** melanoma, antibody, B cell, CD4 T cell, Immunology and Microbiology Section, Immune response, Immunity

## Abstract

The role of B cells and antibodies in anti-tumor immunity is controversial, with both positive and negative effects reported in animal models and clinical studies. We developed a murine B16.F10 melanoma model to study the effects of collaboration between tumor-specific CD4^+^ T cells and B cells on tumor control. By incorporating T cell receptor transgenic T cells and B cell receptor isotype switching B cells, we were able to track the responses of tumor-reactive T and B cells and the development of anti-tumor antibodies in vivo. In the presence of tumor-specific B cells, the number of tumor-reactive CD4^+^ T cells was reduced in lymphoid tissues and the tumor itself, and this correlated with poor tumor control. B cells had little effect on the Th1 bias of the CD4^+^ T cell response, and the number of induced FoxP3^+^ regulatory cells (iTregs) generated from within the original naive CD4^+^ T cell inoculum was unrelated to the degree of B cell expansion. In response to CD4^+^ T cell help, B cells produced a range of isotype-switched anti-tumor antibodies, principally IgG1, IgG2a/c and IgG2b. In the absence of CD4^+^ T cells, B cells responded to agonistic anti-CD40 administration by switching to production of IgG2a/c and, to a lesser extent, IgG1, IgG3, IgA and IgE, which reduced the number of lung metastases after i.v. tumor inoculation but had no effect on the growth of subcutaneous tumors.

## INTRODUCTION

Adaptive immune responses characteristically involve CD4^+^ T cells, CD8^+^ T cells and B cells, all of which may participate in spontaneous and induced responses to cancer in patients [1, 2]. Since cognate interactions between helper CD4^+^ T cells and B cells are required to produce the tumor-specific isotype switched B cells and antibodies detected in many cancer patients [3], the consequences of such interactions are of relevance to our rapidly growing understanding of the anti-tumor immune response.

The use of T cell receptor (TCR) transgenic mice has aided in understanding many of the mechanisms involved in anti-tumor immune responses in preclinical models. Early studies generally focused on direct tumor recognition by CD8^+^ T cells [4–7], while more recently developed models have employed CD4^+^ T cells [8, 9]. One of the best characterized is the Trp-1 model, in which CD4^+^ T cells expressing a transgenic MHC class II (MHCII)-restricted TCR specific for the tyrosinase related protein expressed by B16 melanoma were able to eradicate established tumors in lymphopenic hosts, *via* a direct cytotoxic attack against the B16 melanoma cells, with no requirement for CD8^+^ T cells or B cells [10].

We have developed an alternative preclinical model based on the response of MHCII-restricted TCR transgenic cells to tumor antigen [11]. In contrast to the Trp-1 model, the mechanism of tumor eradication in this model is an IFN-γ-dependent response that requires indirect recognition of tumor antigen presented by host cells. Thus our model mimics a common situation in which tumor antigen-specific CD4^+^ T cells are unable to directly recognize an MHCII-negative tumor. Once again tumor eradiation in immunodeficient hosts requires neither CD8^+^ T cells or B cells [11].

Here we have adapted our transgenic model to the study of B cells in tumor immunity. Despite a substantial body of work, there is as yet no consensus as to whether B cells have a positive or negative effect on tumor clearance [12]. Recent reports showing that immunotherapy with checkpoint inhibitors such as Ipilimumab can activate pre-existing and de novo B cell responses [1], in addition to de novo CD4^+^ T cell responses [13], have served to underline the ongoing clinical relevance of achieving a broader understanding of the role of T-B collaboration in anti-tumor immunity.

Several large-scale clinical studies have suggested that B cells are protective, since B cell infiltration into tumors has been correlated with increased survival of patients with a range of cancers [14–16]. In contrast, the presence of spontaneous serum antibody to tumor-associated antigens (TAAs) is usually either of no prognostic significance or shows a negative association with survival [17, 18]. However generation of antibody responses to TAAs in response to specific immunotherapy can be a positive prognostic indicator [1].

Positive and negative roles of B cells have also been explored in animal models of tumor immunity. T cell priming to tumor antigen is generally enhanced in the absence of B cell antigen presentation [19, 20], and B cells can acquire regulatory functions that negatively influence T cell-dependent anti-tumor immunity [21]. In contrast, pro-inflammatory antibody isotypes have been shown to mediate protection in metastatic disease models [22] but have also been implicated in driving chronic inflammation, which in turn may predispose to malignancy [23].

To examine how collaboration between tumor-specific CD4^+^ T cells and B cells, and the production of isotype switched antibodies to tumor antigens affect tumor growth, we made use of antigen receptor transgenic B cells and CD4^+^ T cells specific for a neo-antigen expressed by the B16 mouse melanoma. By co-transferring CD4^+^ T cells and B cells into tumor-bearing immunodeficient hosts, we determined the effects of B cell antigen presentation and antibody production on tumor protection and the anti-tumor CD4^+^ T cell response. Tumor-specific B cells reduced the number of tumor-reactive CD4^+^ T cells in secondary lymphoid tissues and the tumor itself, but had surprisingly little effect on the CD4^+^ T cell-derived cytokine profile. The absolute number of induced FoxP3^+^ regulatory T cells (iTregs) within the tumor-specific CD4^+^ T cell compartment was unaffected by the presence of B cells, although the B cell-dependent reduction in absolute numbers of CD4^+^ T cells caused iTregs to represent a higher proportion of CD4^+^ T cells. B cells responding to tumor antigen in the presence of CD4^+^ T cell help proliferated, differentiated into germinal center cells and secreted isotype switched anti-tumor antibodies detectable in the serum. In the absence of T cells, B cells activated by anti-CD40 mAb also produced tumor-specific isotype-switched antibodies, which had no effect on the growth of subcutaneous tumors but provided protection in a B16 lung metastasis model.

## RESULTS

To investigate the effects of tumor-specific B cells on tumor eradication by CD4^+^ T cells, we set up a mouse model combining B cell receptor transgenic hen egg lysozyme (HEL)-specific B cells capable of antibody isotype switching (SW_HEL_ mouse model [24]) and TCR transgenic moth cytochrome c (MCC)-specific CD4^+^ T cells (5C.C7 TCR model [25]). Both transgenic mouse lines were bred on a *Rag2^−/−^* background to ensure that they generated only monospecific B and T cells, respectively, and contained no other T or B cells. The B16.F10 melanoma cell line was retrovirally transduced to express a fusion protein containing the relevant HEL and MCC epitopes (designated B16.mHELMCC) [11].

Immunodeficient *Rag*^−/−^ mice were chosen as tumor hosts because of their capacity to support T cell-dependent tumor clearance after adoptive transfer of naive tumor-specific CD4^+^ T cells [11, 26]. This model is the first to combine BCR and TCR transgenic cells for the concurrent assessment of T cell, B cell and antibody responses during the course of a well-defined *in vivo* anti-tumor immune response.

### Effect of B cells on the CD4^+^ T cell response to subcutaneous tumor

**Figure 1:**
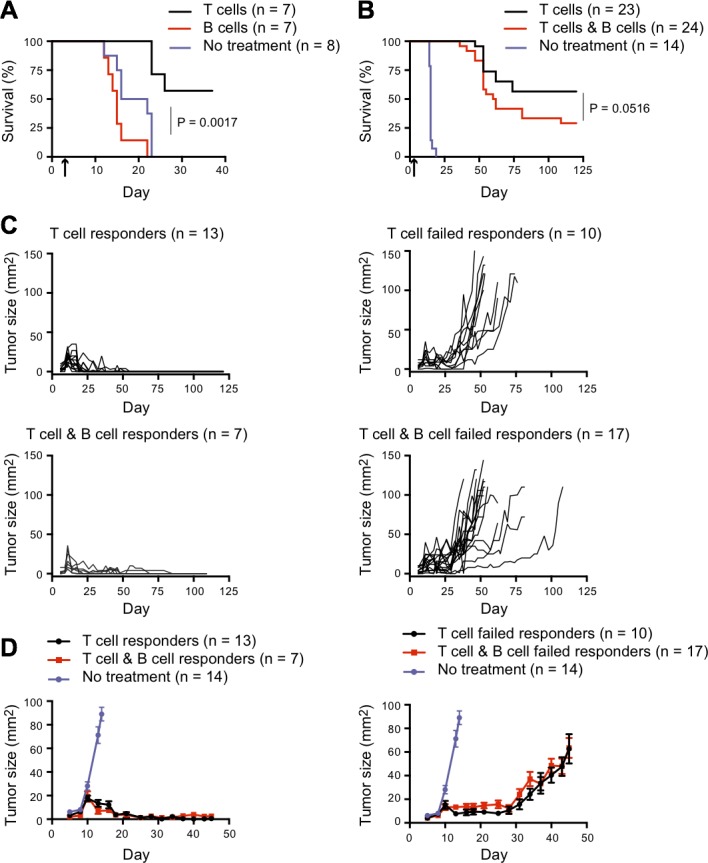
Effect of B cell co-transfer on CD4^+^ T cell-dependent tumor control **A.** *Rag2*^−/−^ mice were injected s.c. in the flank with 0.5×10^6^ B16.mHELMCC tumor cells and adoptively transferred with 0.2×10^6^ naive 5C.C7 T cells or 1×10^6^ naive SW_HEL_ B cells 5 days later (indicated by arrow on graph x-axis). Mice were euthanized when tumors reached 100mm^2^. Kaplan-Meier survival analysis of the three groups; black line: 5C.C7 T cells (*n* = 7), red line: SW_HEL_ B cells (*n* = 7) and purple line: no treatment (*n* = 8). *P* = 0.0017 for comparison between no treatment and T cell groups, while difference between no treatment and B cell groups was not significant as calculated using Log-rank test with Gehan-Breslow-Wilcoxon post-test. (B-D) *Rag2*^−/−^ mice were injected s.c. in the flank with 0.5×10^6^ B16.mHELMCC tumor cells and 3 days later adoptively transferred with 0.2×10^6^ naive 5C.C7 T cells or a combination of 0.2×10^6^ naive 5C.C7 T cells and naïve 1×10^6^ SW_HEL_ B cells (indicated by arrow on graph x-axis). Data are representative of two independent experiments. Mice were euthanized when tumors reached 100mm^2^, with Kaplan-Meier survival analysis shown in **B.** Black line: 5C.C7 T cells (*n* = 23), red line: 5C.C7 T cells plus SW_HEL_ B cells (*n* = 24), purple line: no treatment (*n* = 14). *P* = 0.0516 for comparison between T cell and B cell groups, by Log-rank test with Gehan-Breslow-Wilcoxon post-test. **C.** Individual tumor growth curves separated on the basis of tumor control into long-term responders (tumor-free at 120 days) and failed responders. Top left: 5C.C7 T cell group, responders (*n* = 13). Top right: 5C.C7 T cell group, failed responders (*n* = 10). Bottom left: 5C.C7 T cells plus SW_HEL_ B cell group, responders (*n* = 7). Bottom right: 5C.C7 T cells plus SW_HEL_ B cell group, failed responders (*n* = 17). **D.** Tumor growth (mean±SEM) of responders (left) and failed responders (right) overlaid with control group that received no treatment (purple dot). Note the change in scale, to highlight the early effects on tumor growth.

In a preliminary experiment, adoptive transfer of naive B cells from SW_HEL_ *Rag*2^−/−^ donors had no effect on the growth of subcutaneous B16 tumors, whereas survival was improved in mice adoptively transferred with naive CD4^+^ T cells (100% FoxP3-negative) harvested from 5C.C7 TCR *Rag*2^−/−^ mice (Figure 1A). We next compared tumor growth in mice adoptively transferred with naive 5C.C7 TCR *Rag*2^−/−^ T cells alone *vs* 5C.C7 T cells plus SW_HEL_ B cells. All mice adoptively transferred with CD4^+^ T cells showed highly significant control of tumor growth compared with controls that received no cells (Figure 1B). Mice that received T plus B cells compared with T cells alone showed a trend towards reduced ability to control tumor growth after day 50, although the effect failed to reach statistical significance (*p* = 0.0516) (Figure 1B). Individual tumor growth curves in this experiment showed three patterns: exponential tumor growth in untreated controls, complete tumor clearance with long-term tumor-free survival in a proportion of adoptive hosts of T plus B cells or T cells alone (termed ‘responders’), and early tumor control followed by progressive outgrowth in the remainder of adoptive hosts (termed ‘failed responders’) (Figure 1C and 1D). The latter patterns correspond to those we have previously described in tumor-bearing *Rag*^−/−^ recipients of 5C.C7 TCR *Rag*2^−/−^ T cells [11]. In our previous experiments, we have never observed tumor recurrence in mice that are tumor-free at 120 days, even after regular observation for more than one year, and therefore in the present study, mice that were tumor-free at 120 days were deemed to have mounted a long-term response and were euthanized. Mean tumor growth in responders within the T plus B cell *versus* T cell group was indistinguishable, as was growth in failed responders from the two groups (Figure 1D).

**Figure 2:**
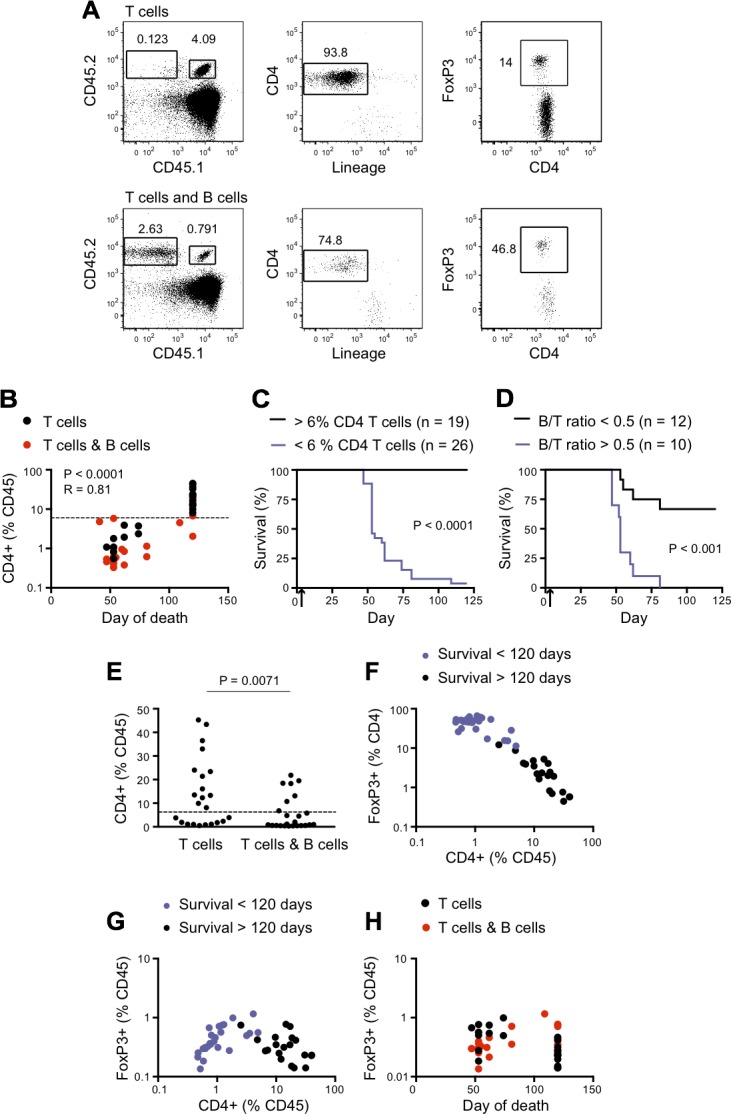
Immune determinants of long-term tumor control For the experiment described in Figure 1B, donor T cell frequencies in the blood were analyzed on day 40. **A.** 5C.C7 T cells were gated as singlet^+^, CD45.1^+^ CD45.2^+^, CD4^+^ and lineage^−^ (lineage cocktail included mAbs against CD11b, Gr1, CD8, NK1.1 and Ter119). iTreg cells were further identified by intracellular FoxP3 expression; the percentage positive is indicated. CD45.2 homozygous SW_HEL_ B cells were identified as CD45.2^+^CD45.1^−^ (tumor bearing hosts were CD45.1 homozygous). Top panel: Recipients of 5C.C7 CD4^+^ T cells alone. Bottom panel: Recipients of 5C.C7 CD4^+^ T cells plus SW_HEL_ B cells. **B.** Frequency of CD4^+^ (% CD45) in the blood on day 40 *vs* day of death. The frequency of CD4^+^ was calculated as a percentage of total CD45.1^+^ cells (excluding B cells), to correct for the presence of B cells in the T plus B cell group. With one exception, all mice tumor free at the completion of the experiment (day 120) had more than 6% CD4^+^ (% CD45) (dashed line) on day 40. Statistical significance was calculated by Spearman correlation. **C.** Kaplan-Meier survival analysis after stratification for > 6% CD4^+^ T cells as a percentage of CD45.1^+^ cells in PBMC on day 40. Black line: > 6% CD4^+^ T cells (*n* = 19). Purple line: < 6% CD4^+^ T cells (*n* = 26). **** *P* < 0.0001 by Log-rank test with Gehan-Breslow-Wilcoxon post-test. **D.** Kaplan-Meier survival analysis after stratification for B cell/CD4^+^ T cell ratio > 0.5 in blood on day 40. Black line: B cells/CD4^+^ T cells < 0.5 (*n* = 12). Purple line: B cells/CD4^+^ T cells > 0.5 (*n* = 10).). *** *P* < 0.001 by Log-rank test with Gehan-Breslow-Wilcoxon post-test. **E.** Frequency of CD4^+^ T cells as %CD45.1 in the blood on day 40 in mice that received either T cells alone or T cells plus B cells. Dashed line represents 6%. Data are representative of two independent experiments. (F, G) Frequency of FoxP3^+^ T cells as percent of CD4^+^ T cells **F.** or percent of CD45.1^+^ cells **G.** in the blood on day 40, plotted relative to frequency of CD4^+^ T cells in the blood on day 40. Black dots: > 120 days survival (*n* = 19). Purple dots: < 120 days survival (*n* = 26). Data are representative of two independent experiments. **H.** Frequency of FoxP3^+^ Tregs of total CD45.1^+^ in blood on day 40, plotted relative to the day of death. Black dots: T cell group. Red dots: T cell plus B cell group. No statistical significance was reached for either group by Spearman correlation. Data are representative of two independent experiments.

To explore the mechanisms underlying the loss of CD4^+^ T cell-dependent tumor control in non-responder mice, we examined several aspects of the CD4^+^ T cell response. Mice in the experiment shown in Figure 1B were bled on day 40 for quantitation of T cell number and detection of FoxP3 as an indicator of conventional CD4^+^ T cell conversion to an iTreg phenotype (Figure 2A). B cells were also quantitated in the T plus B cell group. At day 40, the percentage of CD4^+^ T cells within peripheral blood mononuclear cells correlated with survival (Figure 2B). Only 1 of 26 mice with fewer than 6% circulating CD4^+^ T cells remained tumor free until sacrifice at day 120, compared with all 19 mice with more than 6% CD4^+^ T cells (Figure 2C). The ratio of B cells to CD4^+^ T cells was also predictive of survival, with a higher ratio significantly associated with a worse prognosis (Figure 2D). A significant reduction in the percentage of CD4^+^ T cells was seen in the T plus B cell group when compared with T cells alone (Figure 2E). Although FoxP3^+^ cells expressed as a percentage of CD4^+^ T cells also correlated with survival (Figure 2F), this effect appeared to be secondary to changes in the number of CD4^+^ T cells, as there was no correlation between survival and FoxP3^+^ cells when expressed as a percentage of total (non-B cell) circulating peripheral blood mononuclear cells (Figure 2G, 2H).

**Figure 3:**
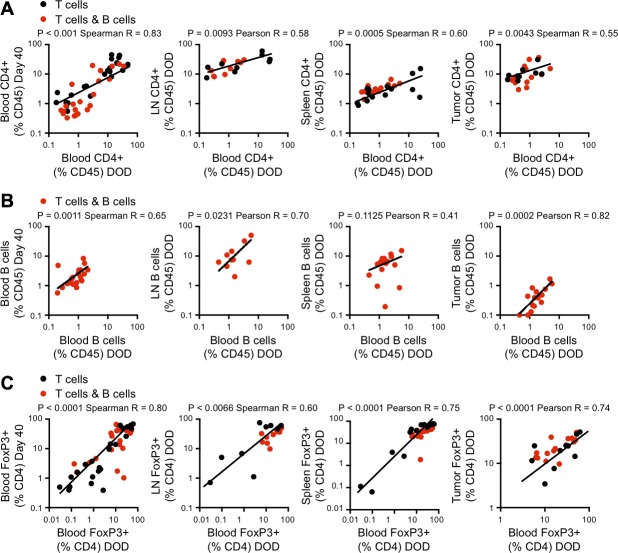
Analysis of immune cell populations in blood, lymph nodes, spleen and tumor In the experiment described in Figure 1B, flow cytometric analysis of blood, lymph nodes, spleen and tumor was performed at the time of euthanasia (when tumors reached 100mm^2^). **A.** Correlation between CD4^+^ T cell frequency in the blood on the day of death (DOD) and on day 40 (as shown in Figure 2) (left panel) and CD4^+^ T cell frequencies in blood and lymph node, spleen and tumor on DOD (right panels). Black dots: 5C.C7 CD4^+^ T cell group. Red dots: 5C.C7 CD4^+^ T cell plus SW_HEL_ B cell group. Log-transformed data. **B.** Correlation between B cell frequency in the blood on DOD and d40 (left panel) and between blood and lymph node, spleen and tumor on DOD (right panels). **C.** Correlation between Foxp3^+^ iTreg frequency as a percent of CD4^+^ T cells in the blood on DOD and d40 (left panel) and FoxP3^+^ (%CD4^+^) in blood and lymph node, spleen and tumor on the DOD (right panels). Black dots: 5C.C7 CD4^+^ T cell group. Red dots: 5C.C7 CD4^+^ T cell plus SW_HEL_ B cell group. For each analysis, data from both groups was pooled, and a D'Agostino-Pearson omnibus normality test was performed prior to correlation analysis to determine the appropriate parametric or non-parametric test. Parametric Pearson correlation coefficients or nonparametric Spearman correlations were used as indicated on each panel.

The percentages of circulating CD4^+^ T cells on day 40 correlated with the percentages in peripheral blood at death (Figure 3A, left panel). Subsequent analysis showed that at the time of euthanasia (when tumor size reached > 100mm^2^ or at day 120), the percentage of peripheral blood CD4^+^ T cells correlated with the CD4^+^ T cell proportions in the spleen and tumor-draining LN, and within the tumor itself in tumor-bearing mice (Figure 3A, right panels). This suggested that the adaptive anti-tumor response had essentially reached equilibrium by day 40. Similar correlations between circulating cell percentages at day 40 and at the time of euthanasia were seen for B cells (Figure 3B) and FoxP3^+^ T cells expressed as a percentage of CD4^+^ T cells (Figure 3C). Taken together, these data suggested that the number of tumor-reactive CD4^+^ T cells at equilibrium was a major determinant of tumor control. While early cognate interactions with tumor-specific B cells reduced the number of CD4^+^ T cells, the total number of iTreg cells was independent of the number of CD4^+^ T cells and B cells and was not predictive of survival.

**Figure 4:**
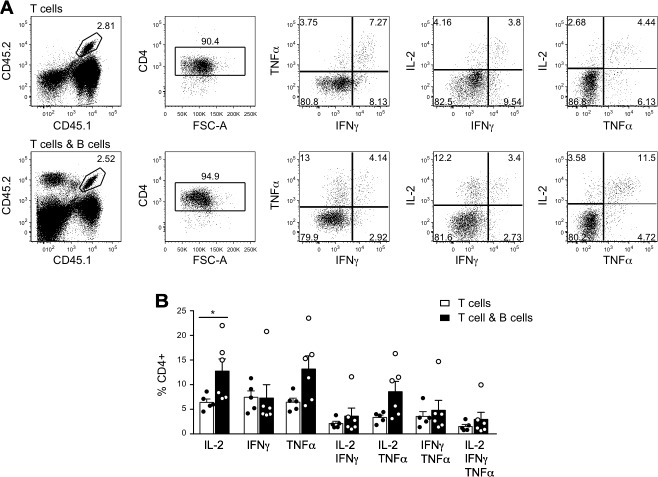
Cytokine production by CD4^+^ T cells in tumor-bearing mice In the experiment described in Figure 1B, 6 mice that received T and B cells and 5 mice that received T cells alone were euthanized as tumors reached critical size on day 52 post tumor cell inoculation. Splenocytes were cultured overnight with MCC peptide and analyzed by flow cytometry for intracellular cytokine production. **A.** Representative dot plots for mice that received 5C.C7 CD4^+^ T cells alone (top panels) or 5C.C7 CD4^+^ T cells plus SW_HEL_^+^ B cells (bottom panels). T cells were gated as CD45.1^+^CD45.2^+^CD4^+^ and analyzed for IL-2, TNFα and IFNγ expression. Frequency of gated events is shown. **B.** Frequency of cytokine producing cells within CD4^+^ T cells. Individual measurements (dots) and mean±SEM of each group are shown. * = *p* < 0.05 by unpaired Student's *t* test.

To explore possible mechanisms of B cell modulation of the CD4^+^ T cell response, we tested cytokine production by splenic T cells in a cohort of T plus B cell *versus* T cell alone recipient mice from the experiment shown in Figure 1B, euthanized with > 100mm^2^ tumors on day 52. Intracellular production of IL-2, TNF and IFNγ by tumor-specific T cells was examined after overnight stimulation with MCC peptide (Figure 4A). Surprisingly, given previous studies suggesting that cognate T-B interactions cause deviation towards a Th2 cytokine profile [27], the frequencies of IL-2-, IFNγ- and TNF-producing cells were not decreased in the T plus B cell group. Indeed, the percentage of IL-2^+^ cells was significantly increased, and there was a trend towards an increase in TNF^+^ cells (Figure 4B). Secreted cytokine production was also analyzed after an overnight culture with a combination of MCC peptide and HEL protein. There was no significant difference between the groups in the levels of IFNγ, TNF, IL-1β, IL-2, IL-3, IL-4, IL-5, IL-6, IL-10, IL-13, IL-17, KC, MCP-1, MIG, MIP-1α and MIP-1β analyzed using a cytometric bead array (CBA) cytokine kit (not shown). The cytokine profile was strongly biased towards Th1, with levels of TNF and IFNγ at least 100-fold those of IL-4, IL-5 and IL-13.

Apart from their reported propensity to induce iTregs, B cells can also develop intrinsic regulatory function [28, 29], which might reduce T cell numbers *in vivo*. Several different phenotypes of regulatory B cells (Bregs) have been reported, including the B10 cell, which regulates anti-tumor responses *via* IL-10 production [21]. We used surface staining against the B10/Breg markers CD5, CD1d and Granzyme B in combination with CD19 and were able to identify cells with a Breg phenotype in wild-type controls but not in tumor-bearing recipients of T and B cells (data not shown). Although we measured IL-10 at levels of 30 pg/ml in culture supernatants (data not shown), there was no difference between cultures with and without B cells and *ex vivo* restimulation of B cells from tumor-bearing mice failed to detect the presence of IL-10 producing B cells (data not shown).

### Effect of CD4^+^ T cells on the B cell response to subcutaneous tumor

**Figure 5:**
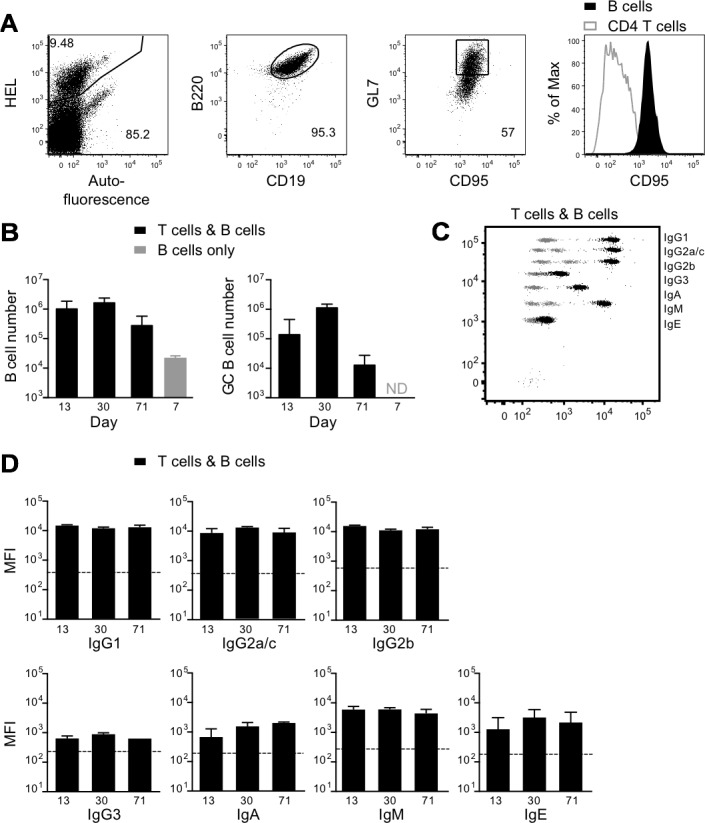
Effect of CD4^+^ T cells on activation, differentiation and antibody production by tumor-specific B cells *Rag2*^−/−^ mice were injected s.c. in the flank with 1×10^6^ B16.mHELMCC tumor cells. On day 3, mice received 1×10^6^ SW_HEL_ B cells plus 0.2×10^6^ 5C.C7 T cells i.v. T cell and B cell numbers and serum antibodies were measured 13, 30 and 71 days later. **A.** Representative dot plots show gating strategy for donor B cells in the spleen. SW_HEL_ B cells were identified as HEL^+^B220^+^CD19^+^ and germinal center SW_HEL_ B cells as HEL^+^B220^+^CD19^+^CD95^+^GL7^+^. Expression of CD95 by germinal center B cells is compared with that of T cells (right panel, T cells gated as HEL^−^CD45.2^+^ within the HEL^−^CD45.2^−^ host, as shown in Figure 4A lower panels). **B.** Black bars: mean±SEM of the number of splenic SW_HEL_ B cells (left) and splenic germinal center (CD95^+^GL7^+^) SW_HEL_ B cells (right) in recipients of B cells and 5C.C7 T cells, on days 13, 30 and 71 post T and B cell transfer (*n* = 4/group). Grey bar: absolute number of splenic SW_HEL_ B cells in s.c. tumor-bearing mice 7 days after transfer of 1×10^6^ 5C.C7 T cells and 5×10^6^ SW_HEL_^+^ B cells lacking expression of the MHCII allele IE required for presentation of the MCC epitope to T cells. Germinal center B cells were not detected (ND) in the B cell only control group. **C.** Representative dot plot depicting serum antibody isotype CBA data from recipients of SW_HEL_ B cells plus T cells (black), overlaid on control CBA plot (grey). **D.** MFI of CBA signals for each antibody isotype on days 13, 30 and 71 post T and B cell transfer (mean±SEM, n≥4/group), with geometric mean of CBA bead only control for each isotype indicated by the dashed line. Data are representative of 2 independent experiments.

To confirm that the HEL-specific B cells were mounting a CD4^+^ T cell dependent response to subcutaneous tumor, we inoculated mice s.c. with B16.mHELMCC cells, adoptively transferred 1×10^6^ naïve SW_HEL_ *Rag*2^−/−^ B cells and 2×10^5^ naive 5C.C7 *Rag*2^−/−^ CD4^+^ T cells on day 3 and characterized splenic B cells by flow cytometry 13, 30 and 71 days later. Transferred SW_HEL_ B cells were identified as HEL-binding B220^+^CD19^+^ cells, and CD95^+^GL7^+^ was used to identify the subpopulation of germinal center cells (Figure 5A). In the presence of cognate T cell help from CD4^+^ T cells, B cells proliferated extensively, with 10^6^ present in the spleen on day 13 post cell transfer, compared with fewer than 2 × 10^4^ in a control group 7 days after transfer of 10^6^ T cells and 5×10^6^ MHCII IE-negative B cells that could not present the MCC epitope in order to attract cognate T cell help. The number of splenic B cells in the T plus B cell group showed no significant decline over the next 2 months (Figure 5B). Germinal center B cells, which were absent from the original naive B cell inoculum, peaked at day 30 and then declined 100-fold by day 71 (Figure 5B). A strong isotype-switched anti-tumor antibody response was produced in response to cognate CD4^+^ T cell help, with large increases in the serum levels of IgG1, IgG2c (the functional equivalent of IgG2a, which is not present in C57BL/6 mice [30]) and IgG2b, and smaller increases in IgG3, IgA and IgE (Figure 5C and 5D). Note that most publications still refer to the C57BL/6 isotype as IgG2a rather than IgG2c, so we have termed the isotype we detected as IgG2a/c throughout.

### Effects of B cells on tumor growth in the absence of T cells

**Figure 6:**
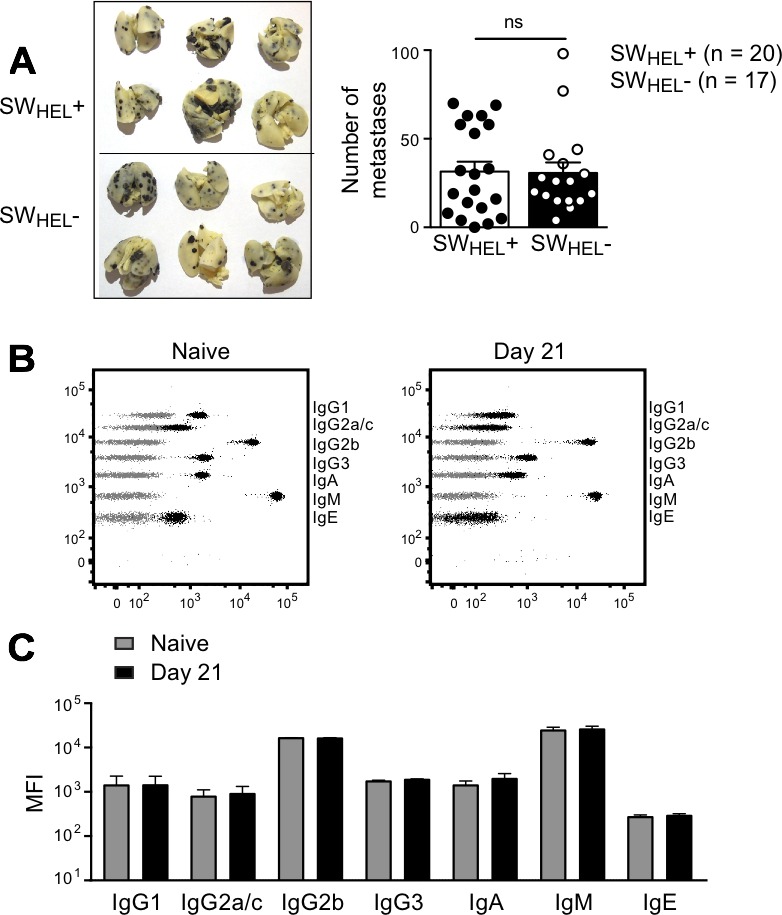
Effect of naive B cells in a lung metastasis model **A.** *Rag*2^−/−^ mice that did or did not express the SW_HEL_ BCR (termed SW_HEL_+ and SW_HEL_-) were injected i.v. with 1×10^6^ B16.mHELMCC tumor cells. Left: Representative photos of lungs from the two groups. Right: Number of metastases on day 21. Each dot represents an individual animal, with the bar representing mean±SEM (*Rag*2^−/−^ SW_HEL_+: *n* = 20, *Rag*2^−/−^ SW_HEL_-: *n* = 17). Data are pooled from 2 independent experiments. ns = not significant by unpaired Student's *t* test **B.** Representative dot plots depicting serum antibody isotype CBA data from *Rag*2^−/−^ SW_HEL_+ mice (black) overlaid on control CBA plot (grey). Sera were collected at the time of death. Left plot: naïve (prior to tumor challenge) SW_HEL_+ mouse. Right plot: SW_HEL_+ mouse after i.v. tumor challenge (time of death: Day 21). **C.** Serum antibody isotype analysis. MFI of CBA signals for each isotype before tumor challenge and on day of death, mean±SEM, n≥7/group. Data are from a single experiment. All isotypes not significantly different by unpaired Student's *t* test.

The experiment shown in Figure 1A indicated that adoptive transfer of naive SW_HEL_ B cells had no effect on the growth of subcutaneous B16.mHELMCC tumors in *Rag*^−/−^ hosts. Since tumor-specific antibody has been shown to protect from lung metastases after i.v. injection of B16 tumor [22], we tested whether SW_HEL_ mice on a *Rag*2^−/−^ background would be more resistant to i.v. challenge with 1×10^6^ B16.mHELMCC tumor than their SW_HEL_-negative *Rag*2^−/−^ littermates that lack all T and B cells. The number of lung metastases was not significantly different in the two groups of mice (Figure 6A). The predominant antibody isotype detected in the serum of naïve SW_HEL_ mice was IgM (Figure 6B), with some spontaneous age-dependent switching to IgG2b and minor amounts of IgG1, IgG3 and IgA (Figure 6B and 6C). Serum antibodies in i.v. tumor-challenged SW_HEL_ mice were similar prior to challenge (naïve state) and at the time of death (day 21), as expected given the absence of T cell help in the *Rag2*^−/−^ mice (Figure 6B and 6C). Thus the presence of naïve tumor-specific B cells secreting IgM did not afford protection from tumor metastasis.

**Figure 7:**
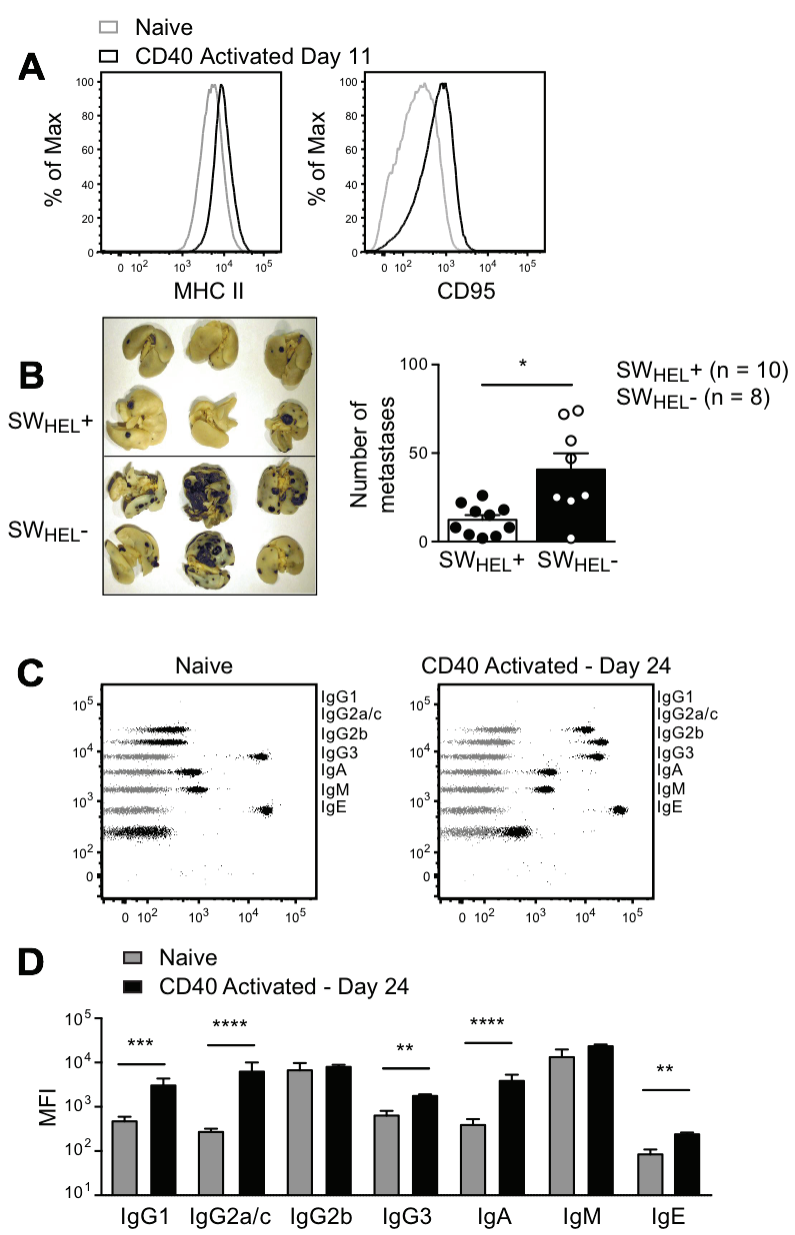
Effect of anti-CD40 activated B cells and isotype switched anti-tumor antibody in a lung metastasis model *Rag*2^−/−^ SW_HEL_+ and *Rag*2^−/−^ SW_HEL_- mice were injected i.v. with 1×10^6^ tumor cells followed by i.p. anti-CD40 on days 3 and 6. **A.** Splenocytes from two anti-CD40 treated *Rag*2^−/−^ SW_HEL_+ mice 11 days after tumor challenge were pooled and stained with B cell activation markers, as indicated. Black histograms: anti-CD40 activated *Rag*2^−/−^ SW_HEL_+ B cells; grey histograms: naive *Rag*2^−/−^ SW_HEL_+ mice. Additional mice were euthanized on day 24, lungs were collected to count metastatic foci and serum was sampled to measure antibody concentration. **B.** Left: Representative photos of lungs from *Rag*2^−/−^ SW_HEL_+ and *Rag*2^−/−^ SW_HEL_- mice. Right: Number of metastases. Each dot represents an individual animal, with the bar representing mean±SEM (SW_HEL_+: *n* = 10, SW_HEL_-: *n* = 8). * = *P* < 0.05 by nonparametric Mann-Whitney test. **C.** Representative dot plots depicting antibody isotype CBA data from *Rag*2^−/−^ SW_HEL_+ mice (black) overlaid on control CBA plot (grey). Left panel: naïve *Rag*2^−/−^ SW_HEL_+ mouse. Right panel: anti-CD40 treated *Rag*2^−/−^ SW_HEL_+ mouse after i.v. tumor challenge (time of death: Day 24). **D.** MFI of CBA signals for each isotype before tumor challenge and on day of death, mean±SEM, n≥9/group. * = *P* < 0.05, ** = *P* < 0.01, *** = *P* < 0.001, **** = *P* < 0.0001 by unpaired Student's *t* test.

To test the potency of isotype-switched tumor-specific antibody in the metastasis model without the confounding influence of tumor-specific CD4^+^ T cells, SW_HEL_ *Rag*2^−/−^ mice were challenged i.v. with tumor cells, followed by 2 i.p. injections of an agonistic anti-CD40 monoclonal antibody to provide helper signals to the B cells. *In vivo* B cell activation in response to anti-CD40 was confirmed by increased expression of MHCII and CD95 on HEL-binding B cells (Figure 7A). The number of lung metastases was significantly decreased in anti-CD40 treated SW_HEL_-positive *Rag*2^−/−^ mice compared to SW_HEL_-negative *Rag*2^−/−^ controls (Figure 7B). The combination of i.v. tumor plus anti-CD40 stimulated a large increase in HEL-specific serum IgG2a/c, with smaller increases in IgG1, IgG3, IgA and IgE and no change in IgG2b or IgM (Figure 7C and 7D). Our finding that IgG2a/c was most prominently associated with the control of B16 lung metastasis is consistent with previous work in which purified exogenous anti-tumor antibody was administered [22].

**Figure 8:**
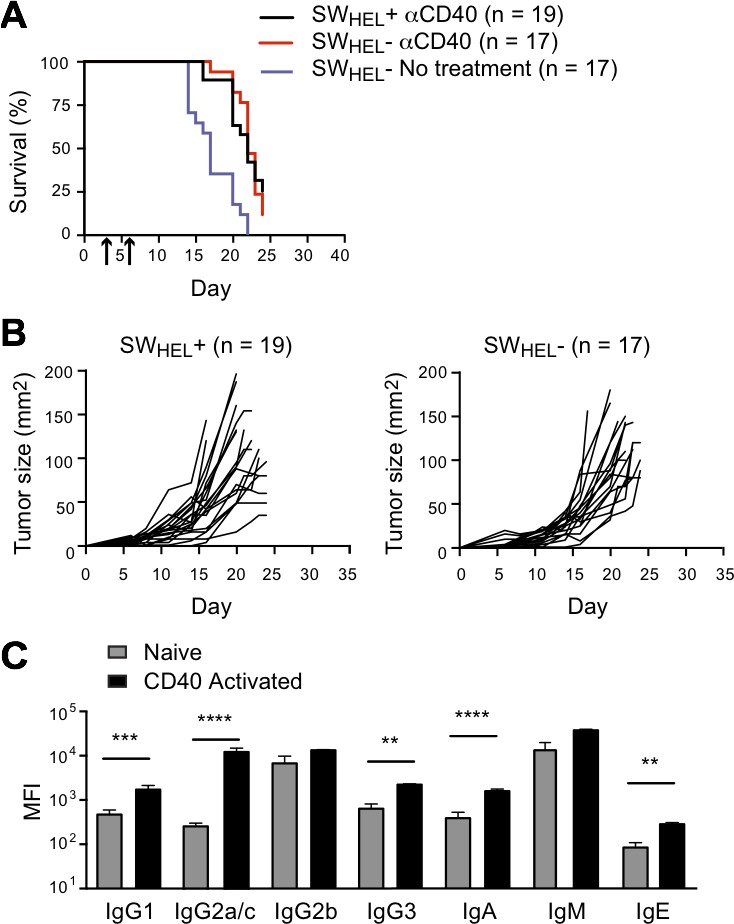
Effect of anti-CD40 activated B cells and isotype switched anti-tumor antibody on growth of subcutaneous tumor *Rag*2^−/−^ SW_HEL_+ and *Rag*2^−/−^ SW_HEL_- mice were injected s.c. with 1×10^6^ tumor cells followed by i.p. anti-CD40 on days 3 and 6. Tumor growth was measured, and serum was collected at the time of death to measure antibody isotypes. **A.** Kaplan-Meier survival analysis. Mice were euthanized when tumors reached 100mm^2^ or at the termination of the experiment on day 24 after tumor cell inoculation. Black line: anti-CD40 antibody treated *Rag*2^−/−^ SW_HEL_+ mice (*n* = 19), red line: CD40 antibody treated *Rag*2^−/−^ SW_HEL_- mice (*n* = 17), with arrows indicating days of anti-CD40 dosing. Purple line: *Rag*2^−/−^ SW_HEL_- mice not treated with CD40 antibody. **B.** Individual tumor growth curves. Left: *Rag*2^−/−^ SW_HEL_+ mice (*n* = 19). Right: *Rag*2^−/−^ SW_HEL_- mice (*n* = 17). **C.** Serum antibody isotypes. MFI of CBA signals for each isotype before tumor challenge (naive) and on day of death (CD40 activated) (mean±SEM, *n* = 10/group). ** = *P* < 0.01, *** = *P* < 0.001, **** = *P* < 0.0001 by unpaired Student's *t* test.

We also tested whether anti-CD40-activated SW_HEL_ B cells could exert a protective effect against s.c. B16.mHELMCC tumors. SW_HEL_ *Rag*2^−/−^ mice and SW_HEL_-negative *Rag*2^−/−^ littermates were inoculated with tumor cells in the flank, followed by anti-CD40 on days 3 and 6. No difference in tumor growth or survival was observed between SW_HEL_-positive and SW_HEL_-negative mice (Figure 8A and 8B). As a control for B cell independent effects of anti-CD40, a second group of SW_HEL_-negative *Rag*2^−/−^ littermates was inoculated with tumor but did not receive anti-CD40. Median survival was reduced from 22 to 17 days, consistent with the previously reported effect of anti-CD40 on macrophages [31]. The antibody response of the anti-CD40-treated SW_HEL_-positive mice to s.c. tumor challenge (Figure 8C) was indistinguishable from that to i.v. challenge (Figure 7D).

**Figure 9:**
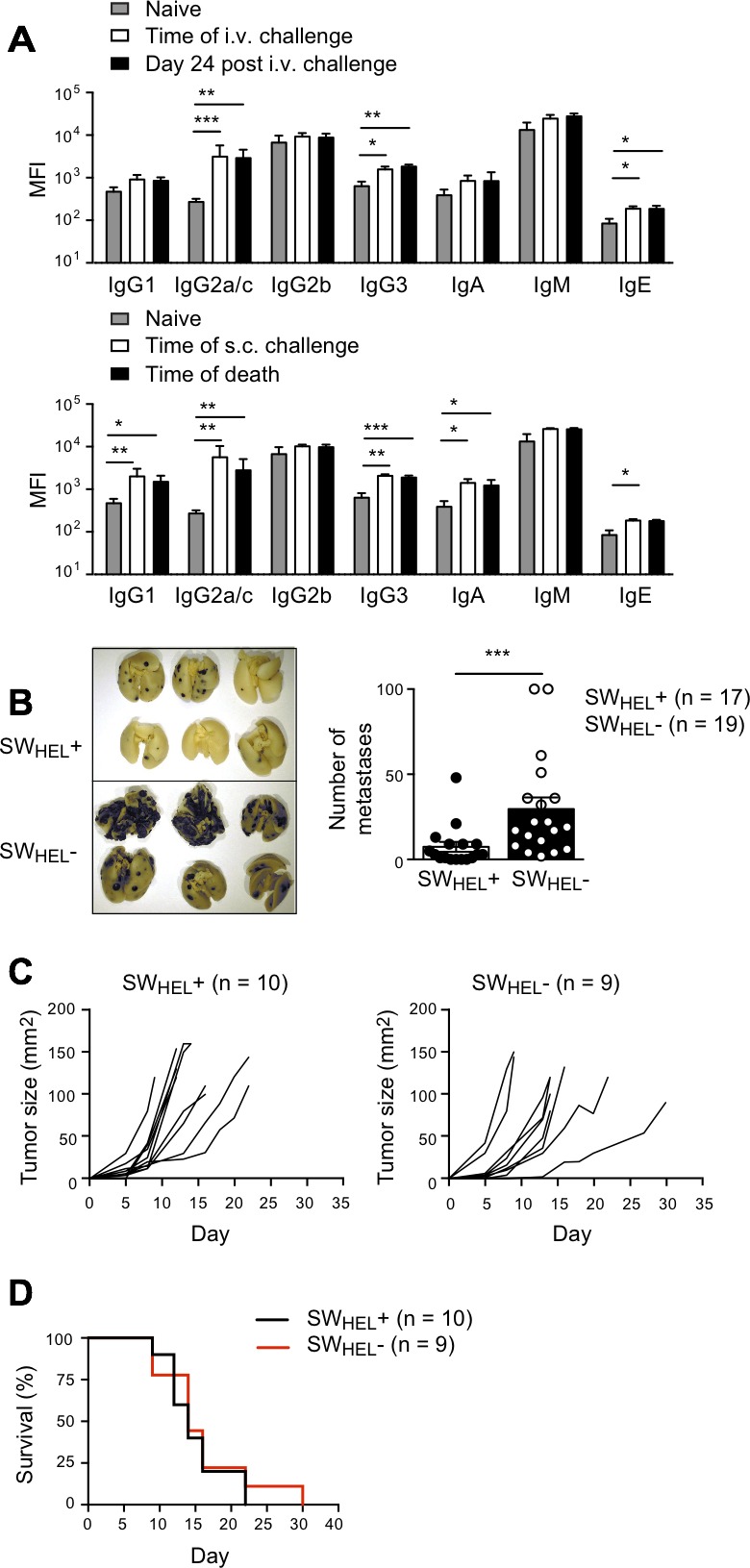
Effect of immunization on B cell control of tumor in subcutaneous and lung metastasis models *Rag*2^−/−^ SW_HEL_+ and *Rag*2^−/−^ SW_HEL_- mice were immunized s.c. with 5×10^6^ irradiated tumor cells emulsified in CFA, followed by i.p. anti-CD40 on days 3 and 6. Two weeks later mice were challenged with 1×10^6^ live tumor cells i.v. or s.c. Mice were euthanized on day 24 after i.v. challenge, or when s.c. tumors reached 100mm^2^. **A.** Serum antibody isotypes. MFI of CBA signals for each isotype before and after immunization, and on day of death (mean±SEM, n≥7/group). Grey bar, naïve *Rag*2^−/−^ SW_HEL_+ mice. White bar, *Rag*2^−/−^ SW_HEL_+ mice at time of i.v. or s.c. challenge (day 14 after immunization). Black bar, *Rag*2^−/−^ SW_HEL_+ mice on day of death). Top: i.v. challenge. Bottom: s.c. challenge. * = *P* < 0.05, ** = *P* < 0.01, *** = *P* < 0.001 by unpaired Student *T* test **B.** Left panel: Representative photos of lungs from *Rag*2^−/−^ SW_HEL_+ and *Rag*2^−/−^ SW_HEL_- mice after i.v. tumor challenge. Right panel: Number of metastases. Each dot represents an individual animal, with the bar representing mean±SEM (SW_HEL_+, *n* = 17, SW_HEL_-, *n* = 19). Data are pooled from 2 independent experiments. *** = *P* < 0.001 by nonparametric Mann-Whitney test. **C.** Individual tumor growth curves after s.c. tumor challenge. Left panel: *Rag*2^−/−^ SW_HEL_+ mice. Right panel: *Rag*2^−/−^ SW_HEL_- mice. **D.** Kaplan-Meier survival analysis after s.c. tumor challenge. Mice were euthanized when tumors reached 100mm^2^. Black line, *Rag*2^−/−^ SW_HEL_+ mice (*n* = 10). Red line, *Rag*2^−/−^ SW_HEL_- mice (*n* = 9).

To rule out the possibility that the failure of antibody to protect against rapidly growing subcutaneous tumors was because the antibody response developed too slowly, we immunized prior to tumor challenge, to generate high levels of isotype switched antibodies. SW_HEL_-positive mice and SW_HEL_-negative *Rag*2^−/−^ littermates were immunized s.c. with irradiated tumor cells emulsified in CFA, followed by anti-CD40 on days 3 and 6. Live tumor challenge was performed s.c. or i.v. on day 14. High levels of IgG2a/c were generated prior to challenge in the SW_HEL_ mice, and these levels were maintained until the mice were sacrificed (Figure 9A). Once again, protection against i.v. but not s.c. challenge was seen (Figure 9B-9D). Taken together, these data suggest that isotype-switched antibodies produced by activated tumor-specific B cells can protect mice against B16 tumor metastases, but have no effect on s.c. tumor growth.

## DISCUSSION

The role of T-B collaboration and production of tumor-specific antibodies in anti-tumor immunity remains controversial. Here we developed a novel pre-clinical model in which the effects of tumor-specific CD4^+^ T cells, B cells and antibodies could be studied under controlled conditions in a well-defined transgenic system. To the best of our knowledge, this is the first report to make use of tumor antigen-specific monoclonal B and T cells to define the effects of T-B collaboration on the anti-tumor response. Our study confirms that tumor-specific B cells can have both positive and negative effects on the growth of transplanted tumor cells in preclinical models, and further defines the immune mechanisms that drive those effects.

We have previously shown that CD4^+^ T cells are capable of eradicating established subcutaneous B16.mHELMCC tumors without any requirement for either CD8^+^ T cells or B cells [11]. In this model, the response of naive tumor-specific CD4^+^ T cells is initiated by tumor-derived antigen presented by host migratory dendritic cells in draining lymph nodes. The T cells then differentiate into Th1-type cells making high levels of IFNγ, which we identified as a major mediator of tumor control [11].

In the current study, the presence of B cells reduced the numbers of tumor-reactive CD4^+^ T cells, which in turn correlated with a reduction in the effectiveness of long-term tumor control, defined as > 120 days tumor-free survival. We found that CD4^+^ T cells in blood, lymph nodes, spleen and the tumors themselves were in equilibrium, and observed a highly significant correlation between the percentages of circulating tumor-specific CD4^+^ T cells at day 40 and at the time of euthanasia. A significant reduction in circulating tumor-specific CD4^+^ T cells was already apparent at day 40 in animals that failed to control tumor growth after an initial phase of stable disease between days 10 and 30. Thus our data indicate that the measurement of tumor-specific lymphocytes in blood can provide a highly relevant correlate of ongoing anti-tumor immunity.

A B cell-dependent reduction in the T cell response to tumor has previously been reported in B cell knockout models [19, 20], but the mechanism is not well understood. Our results indicate that tumor-specific B cells are capable of mediating this effect in the absence of non-specific B cells, suggesting involvement of B cell antigen presentation and/or B cell differentiation in response to specific antigen. Reduction in CD4^+^ T cell numbers and longevity in response to antigen presentation by a combination of B cells and dendritic cells, compared with dendritic cells alone, would be consistent with the findings of Candolfi et al., who used B cell-specific deletion of *Prdm1* to show that B cell differentiation to antibody secretion was not required for their negative effect on T cell-dependent tumor control [32]. The ratio of circulating tumor-specific B to T cells at day 40 was highly predictive of survival in our model, with ratios greater than 0.5 associated with median survival of 53 *versus* > 120 days. Of 11 mice with a B:T ratio of < 0.5, 7 cleared their tumors and remained tumor-free until day 120, while none of those with a ratio > 0.5 survived more than 81 days. These findings raise the possibility that competition between antigen-primed T cells and B cells for access to survival factors within the lymphopenic environment may be involved in the negative effect of B cells on T cell numbers. The major factors required for CD4^+^ T cell proliferation and survival are IL-2 and IL-7. Exogenous IL-2 does not enhance proliferation of CD4^+^ T cells in immunodeficient mice, suggesting that IL-2 is unlikely to be limiting for CD4^+^ T cells under lymphopenic conditions [33]. While IL-7 is important for murine B cell development, very little IL-7 receptor is expressed by primed murine B cells. Thus the identity of factors that might underpin competition between CD4^+^ T and B cells remains unclear.

In addition to measuring the relative numbers of B and T cells, we analyzed cytokine production in tumor-bearing mice that received T and B cells *versus* T cells alone. CD4^+^ T cell priming in the presence of B cells has been reported to drive the response towards a Th2 phenotype [34], which may in turn reduce the effectiveness of T cell-dependent tumor control. However intracellular cytokine staining of splenic CD4^+^ T cells from mice in which tumor escape had occurred indicated an increased rather than decreased proportion of IL-2^+^ T cells in the presence of tumor-specific B cells, with the majority of T cells co-expressing TNF. Production of IFNγ was comparable in the two groups. This bias towards Th1 differentiation is likely due to the high affinity of the 5C.C7 T cells [35], which make IFNγ even under Th2 priming conditions such as use of alum as adjuvant (unpublished observations).

Measurement of cytokine levels in supernatants from overnight culture showed no significant differences in either Th1 or Th2 cytokines between the T plus B *versus* T cell groups, and confirmed the strong Th1 bias. Thus deviation of CD4^+^ T cells away from a type 1 response could not account for tumor escape in our model. We found no evidence of IL-10 production by HEL-specific B cells themselves, and could not identify B cells expressing typical Breg phenotypic markers such as CD5, CD1d and Granzyme B. This is consistent with published results indicating that monoclonal B cells of pre-defined specificity are not recruited into the Breg compartment *in vivo* [36].

Since B cells can induce conversion of CD4^+^ T cells to Foxp3^+^ iTregs [37, 38], we analyzed CD4^+^ T cells for Foxp3 expression at day 40 and the time of euthanasia. We detected iTregs in all recipients of naive FoxP3^−^ 5C.C7 T cells, and their numbers were unaffected by the presence of B cells. In addition, iTregs did not appear to control the effectiveness of CD4^+^ T cell tumor clearance, which was independent of iTreg number. This conclusion is consistent with recent studies reporting that interfering with regulatory T cell function by means of anti-CTLA-4 antibody therapy recruits new T cells into the anti-tumor response, rather than “releasing” already primed cells from Treg-dependent control [39, 40].

We detected very few B cells in the subcutaneous tumors in this model. The percentage of B cells in tumors was approximately 10-fold lower than in blood, while that of CD4^+^ T cells was more than 10-fold higher. Taken together with the linear correlation between B cell numbers in blood and tumor, this suggests that those within the tumor sample likely represent blood contamination. A recent report indicated that B cell infiltration into non-lymphoid tissues may be CD8^+^ T cell dependent, explaining the lack of infiltration in our model [41]. Supporting this interpretation, infiltrating B cells in human tumors are generally associated with a prominent CD8^+^ T cell infiltrate, and the co-location of the 2 cell types correlates with an improved prognosis in many, but not all, tumor types. In contrast, prominent B cell infiltration in the pre-neoplastic phase is highly correlated with progression to frank malignancy in mouse models [23].

In addition to their effects on CD4^+^ T cells, B cells responding to tumor in the presence of T cell help secreted tumor-specific anti-HEL antibody, which we detected in the serum. In tumor-bearing hosts adoptively transferred with both T and B cells, T cell production of IFNγ supported switching to the IFNγ-dependent IgG2a/c isotype. However we also saw large increases in IgG1 and IgG2b, plus lower amounts of IgG3, IgA and IgE. In the absence of T cells, SW_HEL_ B cells could also be activated to secreted isotype switched antibody by administration of anti-CD40 antibody. Compared with CD4^+^ T cell help, anti-CD40 induced an even stronger bias towards IgG2a/c, and protected against lung metastases after i.v. administration of B16.mHELMCC tumor cells. We showed that isotype-switched antibody generated either before or after i.v. B16 tumor administration was protective, indicating that antibody could reduce the growth of already seeded lung metastases. In contrast, we detected no effect of antibody or B cells against subcutaneous tumors in the absence of CD4^+^ T cells, even when the mice had generated optimal isotype-switched antibody responses prior to live tumor cell inoculation. Multiple factors could account for this difference, including enhanced penetration of antibodies into lung metastases compared with skin tumors, and differential presence of FcγR-expressing innate cells at the different sites. In a previous study in which administration of purified anti-tumor antibody synergized with peptide vaccination in a subcutaneous B16 model, FcγR-expressing innate cells in the tumor microenvironment were implicated in early tumor control [42]. Administration of anti-CD40 antibody reduced subcutaneous tumor growth rates in a B cell-independent manner, consistent with the effect on macrophages reported by Beatty et al. [31]. It remains to be determined whether the positive effects of anti-CD40 administration on tumor control in B cell sufficient animals [43] are dependent on macrophages, B cells or both, and whether anti-CD40-mediated B cell activation would alter the outcome of their cognate interactions with T cells in response to specific tumor antigen.

Clinical studies indicate that the presence of spontaneous anti-tumor antibodies is associated with a worse prognosis in some settings [17, 18]. In contrast, de novo antibody responses to immunological interventions such as vaccination with tumor antigen may correlate with an improved prognosis [1]. It is likely that the generation of a potent CD4^+^ T cell-dependent anti-tumor response is the immunological driver behind beneficial clinical responses to cancer, and that the provision of CD4^+^ T cell help to CD8^+^ T cells and B cells generates further positive and negative amplification of anti-tumor immunity, respectively, resulting in eventual control of tumor growth by a combination of CD4^+^ and CD8^+^ T cells, despite the negative effects of B cells.

Our studies suggest that B cell depletion may have beneficial effects when incorporated into a cancer immunotherapy regimen that generates de novo CD4^+^ T cell responses. In the limited number of reports of B cell depletion in patients with non-B cell tumors, it was not administered in conjunction with an immune checkpoint inhibitor, and no major clinical benefit was seen [44, 45]. However an increase in secondary solid tumors was reported after effective anti-CD20 mAb therapy of B cell malignancies [46]. Thus further studies will be needed to test whether B cell depletion has any future role in clinical tumor immunotherapy. In B cell replete settings, de novo appearance of anti-tumor antibodies could usefully serve as a surrogate marker for the generation of a potentially beneficial tumor-specific response to immunotherapy. In addition, it is possible that protective antibody responses may provide a means of decreasing the long-term burden of metastatic tumors, although there is as yet little clinical evidence to support this claim [47]. To date, no clinical protocols have been shown to enhance protective antibody responses while inhibiting B cell-dependent effects on CD4^+^ T cells. Our model provides a basis for the development of such protocols in a controlled setting that yields mechanistic insights.

## MATERIALS AND METHODS

### Mice

All mice were bred and housed under SPF conditions in the Centenary Institute Animal Facility. SW_HEL_ mice expressing the HyHEL10 B cell receptor (BCR) specific for Hen Egg Lysozyme (HEL) were a gift from Robert Brink [24] and were maintained on a C57BL/6 *Rag*2^−/−^ background [48]. 5C.C7 T cell receptor (TCR) transgenic mice specific for Moth Cytochrome C (MCC) [25] were maintained on a B10.BR *Rag*2^−/−^ background (backcrossed to B10.BR for > 10 generations). To ensure histocompatibility between B cells (maintained on C57BL/6) and T cells (maintained on B10.BR), cell donor mice were bred on a [C57BL/6 × B10.BR]F1 background. Intercrosses expressing CD45.2 or CD45.1/CD45.2 were bred from four colonies of *Rag*2^−/−^ mice on C57BL/6 CD45.1, C57BL/6 CD45.2, B10.BR CD45.1 and B10.BR CD45.2 backgrounds (the latter 2 backcrossed onto B10.BR for > 10 generations), allowing us to unequivocally identify each population of adoptively transferred cells. In some experiments, *Rag*1^−/−^ [49] mice on a [C57BL/6 × B10.BR]F1 background (bred in house from C57BL/6 *Rag1*^−/−^ and B10.BR *Rag1*^−/−^ mice (backcrossed for > 10 generations)) were used as tumor hosts instead of [C57BL/6 × B10.BR]F1 *Rag*2^−/−^ mice. We have shown that *Rag1*^−/−^ and *Rag*2^−/−^ F1 immunodeficient host mice behave in an indistinguishable manner in our experimental model. All experiments were performed with the consent of the University of Sydney Animal Ethics Committee, or Sydney Local Health District Animal Welfare Committee.

### Tumor models

The B16.F10 melanoma cell line originally obtained from ATCC was kindly provided by Nikolas Haass. B16.F10 cells were retrovirally transduced to express membrane HELMCC, which consists of HEL protein with residues 64-76 replaced with residues 87-103 of MCC, fused at the C-terminus to the transmembrane and cytoplasmic domains of H-2K^b^ [50]. Transduced tumor cells were screened by flow cytometry and cloned to select a stable line designated B16.mHELMCC, which was used for all experiments [11].

Viable B16.mHELMCC tumor cells were injected either intravenously (i.v.) *via* the tail vein or subcutaneously (s.c.) in the flank. For i.v. tumor cell inoculation, additional test mice were inoculated and euthanized between days 20 and 24 to assess the size of lung metastases and determine the optimal timepoint to euthanize the experimental groups. After euthanasia, mice were perfused with saline solution, and lungs collected in Fekete's solution. The number of metastases was counted visually in a blinded fashion [51]. When the number of metastases was > 100, a score of 100 was given. Subcutaneous tumors were measured in a blinded fashion 2-3 times per week with digital calipers. Mice were euthanized when the tumor area reached 100mm^2^.

For immunization of SW_HEL_ mice, 5×10^6^ irradiated B16.mHELMCC cells (3500cGy) emulsified in complete Freund's adjuvant (CFA) were injected s.c. in one flank. Two intraperitoneal (i.p.) injections of anti-CD40 (FGK45, 25μg/injection) were given on days 3 and 6, and mice were challenged i.v. or s.c. with live tumor cells on day 14. The site of s.c. challenge was the flank opposite to the immunization site.

### Adoptive cell transfer and flow cytometry analysis

For adoptive transfer, splenic SW_HEL_ B cells and TCR transgenic T cells from pooled lymph nodes were co-transferred at a 5:1 ratio (1×10^6^ B cells, 0.2×10^6^ T cells). For flow cytometric analysis of blood, lymph node, spleen and tumor, samples were prepared as described previously [11]. The following monoclonal Abs were used to stain cells: anti-CD4 (RM4-5), anti-CD8 (53-6.7), anti-CD11b (M1/70), anti-NK1.1 (PK136), anti-CD45 (30-F11), anti-CD95 (Jo2), anti-Ly-77 (GL7), anti-MHCII (M5/114.15.2), anti-Ter119 (TER 119) and anti-B220 (RA3-6B2) obtained from BD Biosciences (Franklin Lakes, NJ, USA); anti-CD1d (1B1), anti-CD5 (53-7.3), anti-CD19 (6D5), anti-CD45.2 (104), anti-CD45.1 (A20), anti-Gr1 (RB6-8C5) and anti-Granzyme B (GB11) obtained from BioLegend (San Diego, CA, USA). All antibodies were directly conjugated with FITC, PE, allophycocyanin, Pacific Blue or cyanin conjugates PE-Cy7, PerCP-Cy5.5 or allophycocyanin-Cy7. Non-specific binding to Fc receptors blocked using anti-CD16/32 purified in house from the 2.4G2-hybdridoma. Intracellular staining of FoxP3 was performed using a murine FoxP3 staining kit and anti-Foxp3 mAb (FJK-16s) from eBioscience. SW_HEL_ B cells were detected with HEL protein conjugated to Alexa647, kindly provided by Chris Jolly. Samples were analyzed on LSR-II, Fortessa and FACSCanto BD flow cytometers.

### Cytokine measurements

For intracellular cytokine detection, 2 × 10^7^ splenocytes were plated at 1 × 10^7^ cells/mL in 12 well plates in tissue culture medium containing 10μM MCC peptide and 500ng/mL HEL protein. After 2 hours incubation at 37°C, Brefeldin A (Sigma) was added to a final concentration of 5ng/mL and cells were further cultured for 16 hours. Cells were then washed and stained for cell surface CD45.1, CD45.2 and CD4, followed by intracellular staining with mAbs against IL-2 (JES6-5H4) and IFNγ (XMG1.2) from BD Biosciences, and anti-TNFα (MP6-XT33) from BioLegend. For measurement of cytokines in culture supernatant, splenocytes were plated at 1 × 10^6^ cells/well in 96 well plates in tissue culture medium containing 10μM MCC peptide and 500ng/mL HEL protein. Supernatants were collected after 24 hrs incubation at 37°C and cytokines measured using a CBA cytokine kit (BD, 562246)

### Mouse immunoglobulin isotyping kit

For relative measurement of serum antibody isotypes, a CBA Mouse Immunoglobulin Isotyping Kit (BD, 550026) was used according to manufacturer's instructions. 1μL of serum was used in most assays except when samples were titered to generate dose-response curves for each isotype, allowing estimation of fold concentration changes based on the mean fluorescence intensity (MFI) values. The isotyping kit included monoclonal antibody R19-15, which recognizes both the IgG2a isotype present in most mouse strains and the functionally equivalent IgG2c isotype present in C57BL/6 mice [30], and therefore expressed by SW_HEL_ positive mice. Since most publications still refer to the C57BL/6 isotype as IgG2a rather than IgG2c, we have termed the isotype we detected as IgG2a/c throughout.

### Statistics

All analyses were performed using GraphPad Prism Software. For experiments with 2 groups, Mann-Whitney (nonparametric) or unpaired Student's t (parametric) tests were used. Data are shown as mean ^+^/− SEM. Kaplan-Meier survival curves were analyzed using Log-rank (Mantel-Cox) and Gehan-Breslow-Wilcoxon tests. Pearson correlation coefficients were used for parametric data and Spearman's rank correlation coefficient was used for non-parametric data. A D'Agostino-Pearson omnibus normality test was performed prior to correlation analysis to determine the appropriate parametric or non-parametric test.
